# Sensitive LC-MS/MS Method for the Quantification of Macrocyclic Gα_q_ Protein Inhibitors in Biological Samples

**DOI:** 10.3389/fchem.2020.00833

**Published:** 2020-09-24

**Authors:** Markus Kuschak, Jonathan G. Schlegel, Marion Schneider, Stefan Kehraus, Jan H. Voss, Alexander Seidinger, Michaela Matthey, Daniela Wenzel, Bernd K. Fleischmann, Gabriele M. König, Christa E. Müller

**Affiliations:** ^1^PharmaCenter Bonn, Pharmaceutical Institute, Pharmaceutical & Medicinal Chemistry, University of Bonn, Bonn, Germany; ^2^Institute of Pharmaceutical Biology, University of Bonn, Bonn, Germany; ^3^Institute of Physiology I, Life and Brain Center, Medical Faculty, University of Bonn, Bonn, Germany; ^4^Department of Systems Physiology, Medical Faculty, Ruhr University Bochum, Bochum, Germany

**Keywords:** drug levels, FR900359, Gq inhibitor, LC-MS/MS, preclinical experiments, quantification, quantitative analysis, YM-254890

## Abstract

The cyclic depsipeptide FR900359 (FR) isolated from the plant *Ardisia crenata* and produced by endosymbiotic bacteria acts as a selective Gq protein inhibitor. It is a powerful tool to study G protein-coupled receptor signaling, and has potential as a novel drug for the treatment of pulmonary diseases and cancer. For pharmacokinetic studies, sensitive quantitative measurements of drug levels are required. In the present study we established an LC-MS/MS method to detect nanomolar concentrations of FR and the structurally related natural product YM-254890 (YM) in biological samples. HPLC separation coupled to ESI-QTOF-MS and UV-VIS detection was applied. For identification and quantification, the extract ion chromatogram (EIC) of M+1 was evaluated. Limits of detection (LOD) of 0.53–0.55 nM and limits of quantification (LOQ) of 1.6–1.7 nM were achieved for both FR and YM. This protocol was subsequently applied to determine FR concentrations in mouse organs and tissues after peroral application of the drug. A three-step liquid-liquid extraction protocol was established, which resulted in adequate recovery rates of typically around 50%. The results indicated low peroral absorption of FR. Besides the gut, highest concentrations were determined in eye and kidney. The developed analytical method will be useful for preclinical studies to evaluate these potent Gq protein inhibitors, which may have potential as future drugs for complex diseases.

## Introduction

**Figure 1:**
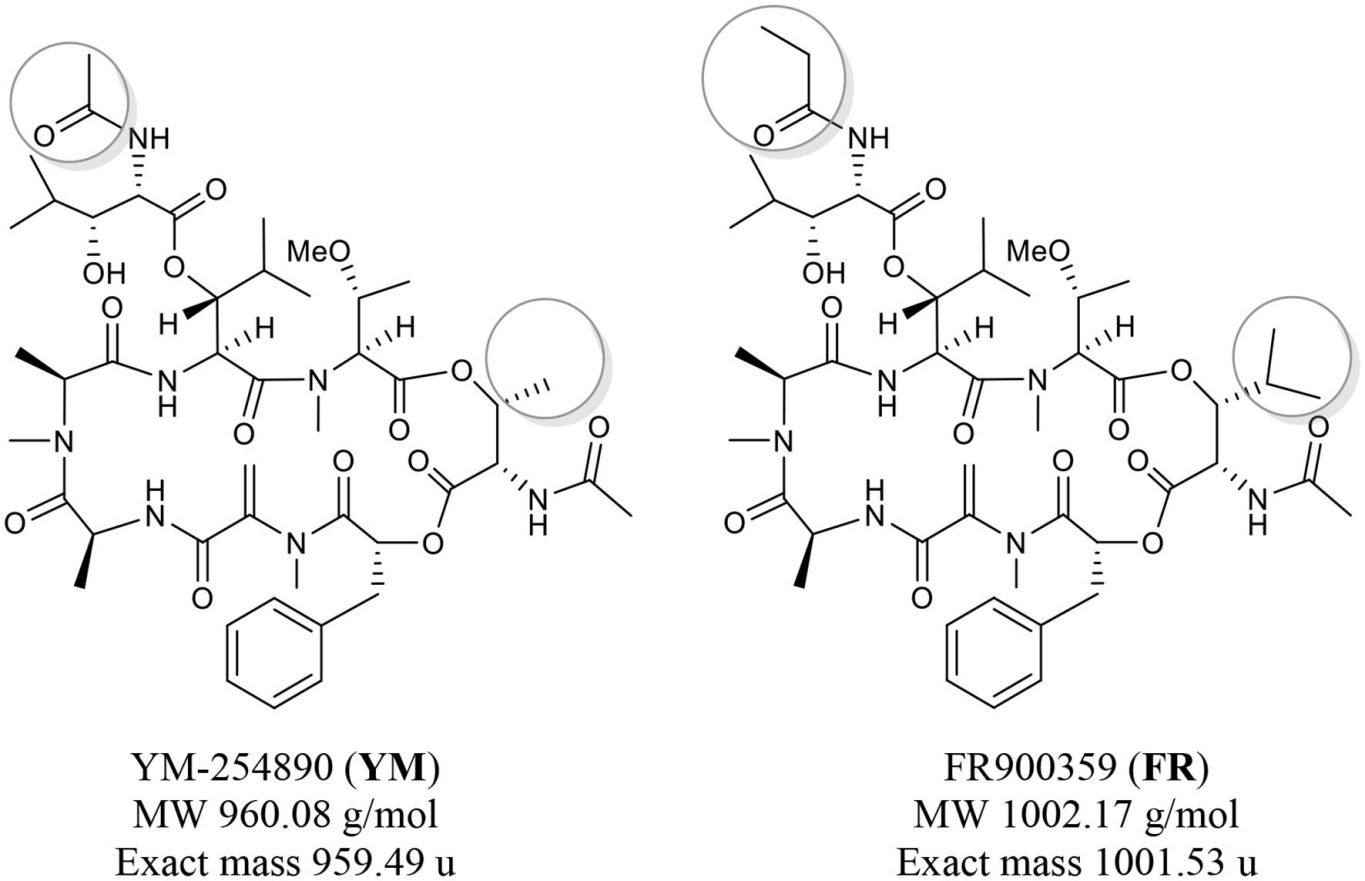
Chemical structures of the macrocyclic depsipeptides YM-254890 (left) and FR900359 (right).

The structurally related natural macrocyclic depsipeptides, YM-254890 (YM) and FR900359 (FR, see Figure 1 for structures) have been described as potent and selective inhibitors of G_q_ proteins (Takasaki et al., 2004; Schrage et al., 2015). YM is synthesized by *Chromobacterium* sp. (Taniguchi et al., 2003) while FR has been isolated from the leaves of the ornamental plant *Ardisia crenata* Sims (Fujioka et al., 1988) and is produced by the bacterial endophyte *Candidatus Burkholderia crenata* (Carlier et al., 2016). The genome analysis of *Candidatus Burkholderia* *crenata* reveals that secondary metabolism may be a key function for the *Ardisia crenata* leaf nodule symbiosis (Carlier et al., 2016). The structure of both depsipeptides differs only in two residues, resulting in a higher lipophilicity of FR as compared to YM: FR contains a propionyl instead of an acetyl residue and an isopropyl instead of a methyl group (Figure 1, highlighted in gray circles). The total synthesis of both compounds and several analogs was accomplished by a multi-step process (Xiong et al., 2016).

As YM and FR represent the only available non-protein entities with a highly specific inhibitory activity toward heterotrimeric G proteins, interest in these compounds within the scientific community has been immense (Chua et al., 2017; Kamato et al., 2017; Kostenis et al., 2020; Zhang et al., 2020). Binding of YM and FR to the Gα_q_ protein subunit is believed to prevent nucleotide exchange of GDP for GTP which keeps the heterotrimeric G protein arrested in its inactive state (Nishimura et al., 2010; Schrage et al., 2015). Inhibition of G_q_ signaling has been investigated in several disease models, and FR in particular, due to its long residence time (Kuschak et al., 2020), was found to be a promising drug candidate for the treatment of complex diseases such as asthma (Matthey et al., 2017), hypertension (Meleka et al., 2019), metabolic syndrome (Klepac et al., 2016), and cancers (Onken et al., 2018; Annala et al., 2019; Lapadula et al., 2019).

The aim of this study was the development of a sensitive and accurate method for the quantitative determination of FR and YM in different animal tissues, which is crucial for preclinical studies. We developed a three-step liquid-liquid extraction (LLE) method along with an LC-MS/MS protocol to detect and quantify nanomolar concentrations of FR and YM in mouse tissues and blood serum.

## Materials and Methods

### Chemicals

FR was isolated as previously described (Schrage et al., 2015). YM was purchased from Wako Chemicals GmbH (Neuss, Germany) with a declared purity of >95%. Stock solutions (1 mM) were prepared in dimethylsulfoxide and stored at −20° C. Dilutions were generated by appropriate dilution of the corresponding stock solutions with methanol/water (1:1) containing 2 mM ammonium acetate and 0.1% formic acid. Chromatography solvents and samples were prepared with LCMS grade methanol, MS grade formic acid (VWR, Leuven, Belgium) and deionized water. Ammonium acetate was purchased from Sigma-Aldrich (Schnelldorf, Germany).

### Preparation of Standard Solutions for Calibration

FR and YM were weighed and dissolved in methanol to create stock solutions of each compound. Subsequently, nine dilutions were prepared from each of the stock solutions to create standard calibration curves with concentrations ranging from 0.5 nM to 10 nM (YM) or from 0.5 nM to 20 nM (FR). Limits of detection (LOD) and limits of quantification (LOQ) were determined by measurement of calibration curves using the developed LC-MS/MS method (see section Quantitative analysis of FR and YM). For quantitative analysis in tissue samples, a calibration curve for FR was determined using 10 standard dilutions ranging from 10 to 2,000 nM.

### Extraction and Quantification of FR From Mouse Tissue Samples

Animal experiments were approved by the local ethics committee and carried out in accordance to the guidelines of the German Animal Welfare Act with approval by the local government authorities (Landesamt für Natur, Umwelt und Verbraucherschutz Nordrhein-Westfalen, NRW, Germany).

A single dose of FR (0.2 mg in 200 μL 1:10 DMSO in 0.9% NaCl) was applied orally to CD-1 mice by a stomach tube. Animals were sacrificed after ~1.25 h and organs as well as plasma were harvested and snap-frozen. FR was extracted by a 3-step liquid-liquid extraction (LLE) method, and quantified by the developed LC-MS/MS protocol (see section Quantitative Analysis of FR and YM).

Blood plasma samples were diluted 1:10 in methanol containing 2 mM ammonium acetate and 0.1% formic acid, and centrifuged at 15,000 g for 20 min at room temperature (step 1). Organic solvent was removed from the supernatant at 200 mbar and 40° C by a SpeedVac (Thermo Fisher Scientific, MA, USA), The residual pellet was dissolved in 100 μl methanol and subjected to LC-MS/MS analysis.

Solid tissue samples (250 mg) were mixed with 1 ml methanol containing 2 mM ammonium acetate and 0.1% formic acid (step 1). Samples were subsequently homogenized in a TissueLyzer (Qiagen, Venlo, Netherlands) for 8 min at 50 strokes/min and centrifuged at 15,000 g and 4° C for 15 min. The supernatant was collected, and the extraction procedure was repeated twice by using pure methanol for the subsequent extractions (step 2–3). The collected extracts were centrifuged at 15,000 g for 30 min. The supernatant was transferred to a SpeedVac and the organic solvent was removed at 200 mbar and 40° C. The residual pellet was dissolved in 100 μl of methanol, vortexed, and centrifuged for 5 min at 15,000 g; the resulting supernatant was subjected to LC-MS/MS analysis.

To determine the recovery rate of FR, organs of non-treated mice were extracted using an extraction buffer spiked with 100 nM FR. The recovery rate was calculated by comparing the measured concentration with the expected concentration (for details see Supporting Information).

## LC-MS/MS Instrumentation and Analytical Conditions

### Quantitative Analysis of FR and YM

Chromatographic separation was performed by HPLC (Dionex Ultimate 3000, Thermo Fisher Scientific, MA, USA) equipped with an integrated variable wavelength detector coupled to a micrOTOF-Q mass spectrometer (Bruker, MA, USA) with an electrospray ion source. An EC50/2 Nucleodur C18 Gravity 3 μm column (Macherey & Nagel, Dueren, Germany) was used for chromatographic separation. Nitrogen was supplied by a high purity generator (Parker G4600E, Parker Hannifin Manufacturing ltd., Gateshead, UK) and used as carrier gas. The sample solution (5 μl) was injected at a flow rate of 0.3 mL/min. The two mobile phases were A (40% aq. methanol containing 2 mM ammonium acetate and 0.1% formic acid) and B (methanol, 2 mM ammonium acetate, 0.1% formic acid). The run started with 100% A. After 1 min, a gradient was started reaching 100% eluent B within 9 min. Then, the column was flushed for 5 min with solvent B. Positive full scan MS was recorded from 200 to 1,500 m/z. The extract ion chromatogram (EIC) of 1002.54 ± 0.01 m/z was used for the identification and quantification of FR by the QuantAnalysis program (Bruker, MA, USA). An EIC of 960.49 ± 0.01 m/z was employed for the identification and quantification of YM.

### Data Analysis

If not otherwise indicated, three independent experiments were performed. Data points in graphs are shown as means ± standard error of the mean (SEM). Data analysis and plotting was performed using GraphPad PRISM, Version 7.0 (GraphPad, San Diego, CA, USA).

## Results

### Development and Validation of a Sensitive Analytical Method

**Figure 2:**
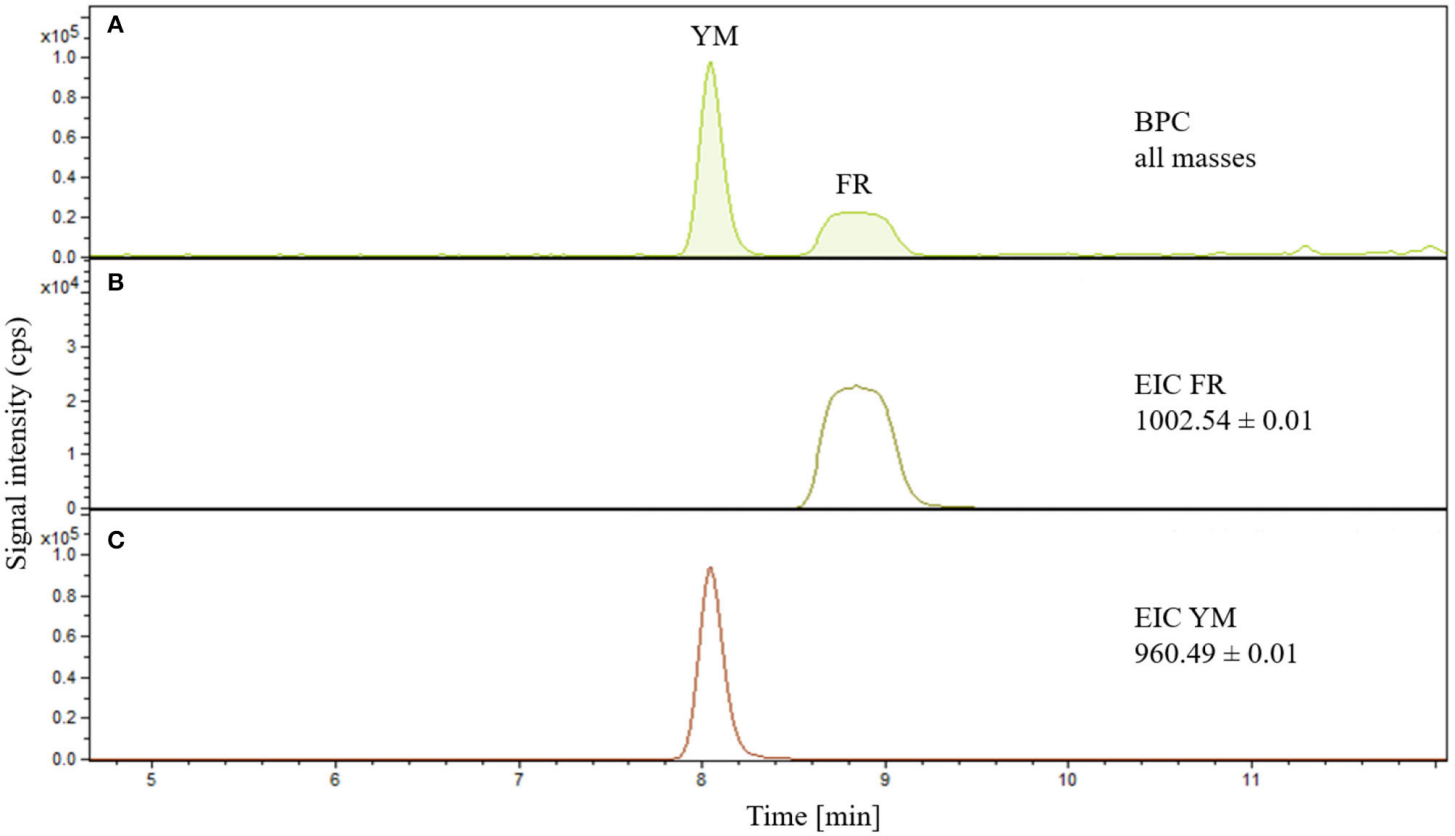
**(A)** Base peak chromatograms of FR900359 and YM-254890 simultaneously detected. **(B)** EIC of FR900359, m/z ratio: 1002.54 ± 0.01. **(C)** EIC of YM-254890, m/z ratio 960.49 ± 0.01.

Due to the close structural similarity and the high lipophilicity of FR and YM, their chromatographic separation turned out to be a major challenge. Initial experiments utilizing a neutral, a basic or an acidic buffer all resulted in peak broadening. Also, employing various reversed phase columns (Phenomenex® Hydro-RP, Max-RP, Polar-RP, and Fusion-RP) did not solve the problem. Finally, an EC50/2 Nucleodur C18 Gravity 3 μm column and a water/methanol-based gradient elution (see section Quantitative analysis of FR and YM) resulted in separation of YM and FR providing baseline-separated peaks for both molecules (Figure 2A).

The established method was validated by determination of linearity, precision, accuracy, limit of detection (LOD), and limit of quantification (LOQ) for both FR and YM (summarized in Table 1 and Supplementary Table 1). The results of intra-day and inter-day precisions and accuracies were calculated by evaluating compound concentrations ranging from 0.5 to 20 nM. The results were within the acceptable limits set by the FDA guideline “Bioanalytical Method Validation Guidance for Industry” (05/24/18). For FR, an LOD of 0.553 nM and an LOQ of 1.68 nM, and for YM an LOD of 0.525 nM and an LOQ of 1.59 nM were determined. The calibration curve for both FR and YM showed a linearity of ≥ 0.99, up to the highest tested concentrations of 2,000 nM (see Supplementary Figure 1).

**Table 1:** Validation of LC/MS-MS method for the quantitative analysis of FR and YM.

**Parameters**	**YM-254890**	**FR900359**	**Acceptable range^a^**
**Regression equation**	*y* = 1.040 x + 0.004211	*y* = 1.003 x−0.008427	
Linearity (*R*^2^; *n* = 3)	0.9939	0.9988	
LOD (nM)	0.5245	0.5532	
LOQ (nM)	1.590	1.676	
**Accuracy (recovery** **±** **standard deviation (SD);** ***n*** **=** **3), [%]**			
0.5 nM	87.8 ± 0.2	91.6 ± 0.1	not applicable^b^
1 nM	103.6 ± 0.1	84.7 ± 0.2	not applicable^b^
2 nM	103.6 ± 0.4	101.7 ± 0.2	85–115
4 nM	102.3 ± 0.3	101.5 ± 0.1	85–115
6 nM	105.7 ± 0.5	100.7 ± 0.2	85–115
8 nM	102.0 ± 0.7	100.9 ± 0.4	85–115
10 nM	98.7 ± 0.4	95.3 ± 0.1	85–115
20 nM	nd^c^	101.3 ± 0.4	85–115
**Precision (relative standard deviation (RSD);** ***n*** **=** **3), [%]**			
0.5 nM	52.4	29.9	not applicable^b^
1 nM	18.2	20.4	not applicable^b^
2 nM	10.3	8.5	<15
4 nM	7.6	0.9	<15
6 nM	6.0	3.3	<15
8 nM	6.6	4.8	<15
10 nM	4.5	1.3	<15
20 nM	Nd	2.2	<15

a *According to the FDA guideline “Bioanalytical Method Validation Guidance for Industry”*.

b *Concentration below LOQ*.

c *nd, not determined*.

**Figure 3:**
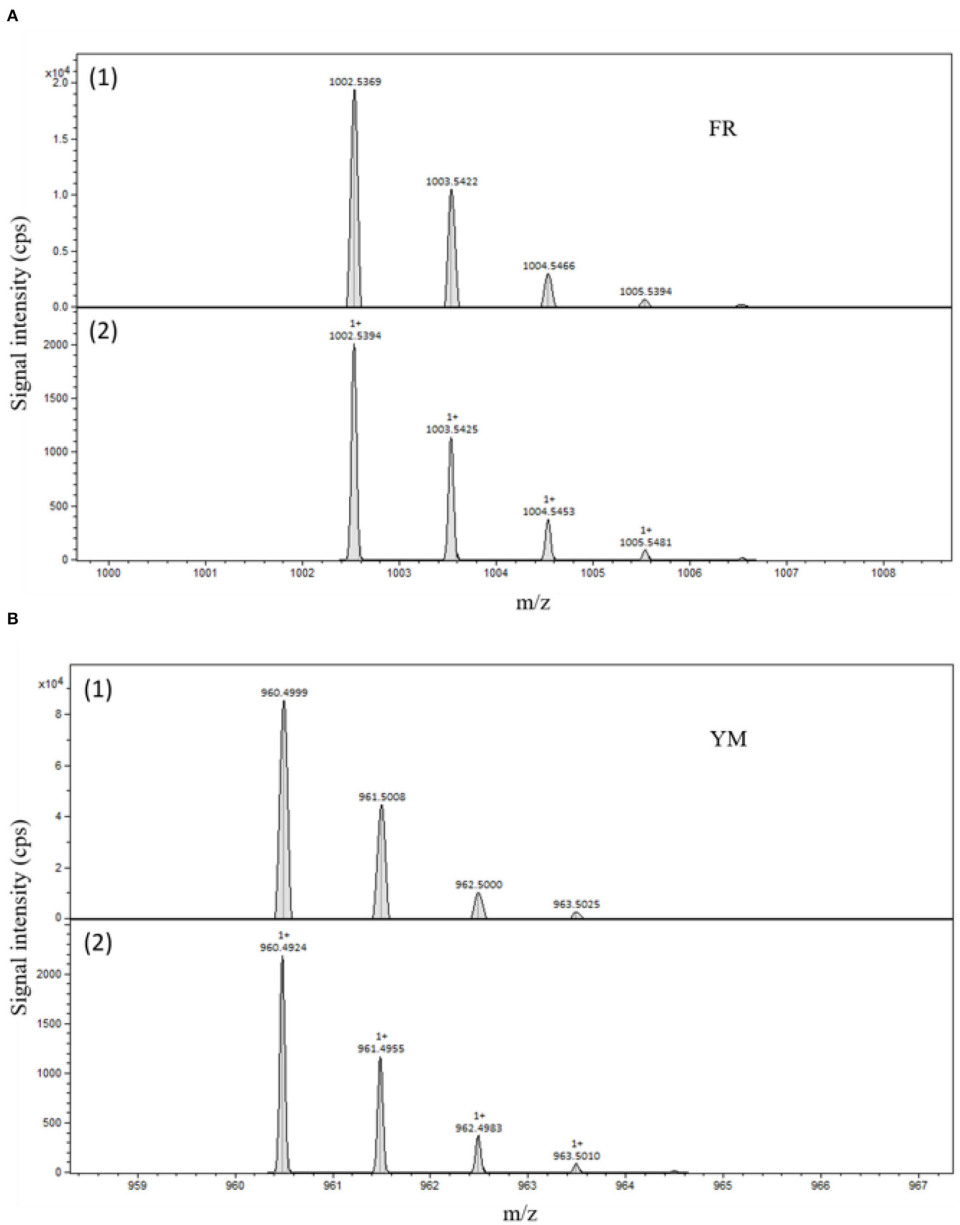
**(A)** (1) Mass spectrum of FR900359 [x axis: m/z range 999.8–1008.6; y axis: signal intensity in counts per second (cps)]; **(A)** (2) Calculated mass isotope pattern of FR900359. **(B)** (1) Top: mass spectrum of YM-254890 [x axis: m/z range 958.4–967.2; y axis: signal intensity in counts per second (cps)]; **(B)** (2) Calculated mass isotope pattern of YM-254890.

Specificity was achieved by using an EIC of 1002.54 ± 0.01 mass-to-charge ratio (m/z) for FR (Figure 2B) and 960.49 ± 0.01 m/z for YM (Figure 2C). Detected peaks correspond to the calculated m/z ratios considering the isotopic distribution (see Figure 3).

### Quantitative Determination of FR in Mouse Serum and Tissues

Next, we applied the developed sensitive analytical method to determine tissue concentrations of FR after peroral application in mice.

As a first step, we had to develop an accurate liquid-liquid extraction method to reliably extract subnanomolar concentrations of FR from mouse tissues while maintaining an acceptable recovery rate. In our first trials, we studied the addition of other macrocyclic drugs as internal standards, including erythromycin and cyclosporin. This failed, however, due to irreproducible results in the presence of FR. FR possibly interacts with these macrocyclic compounds thereby disturbing their extraction and quantification. The best results were achieved using a 3-step liquid-liquid extraction method (for details see section Extraction and Quantification of FR from Mouse Tissue Samples). Utilizing this method, a recovery rate for FR of ~50% on average was obtained (see Supplementary Figure 2). The same method was also used for the extraction of YM and found to be similarly efficient (not shown).

**Figure 4:**
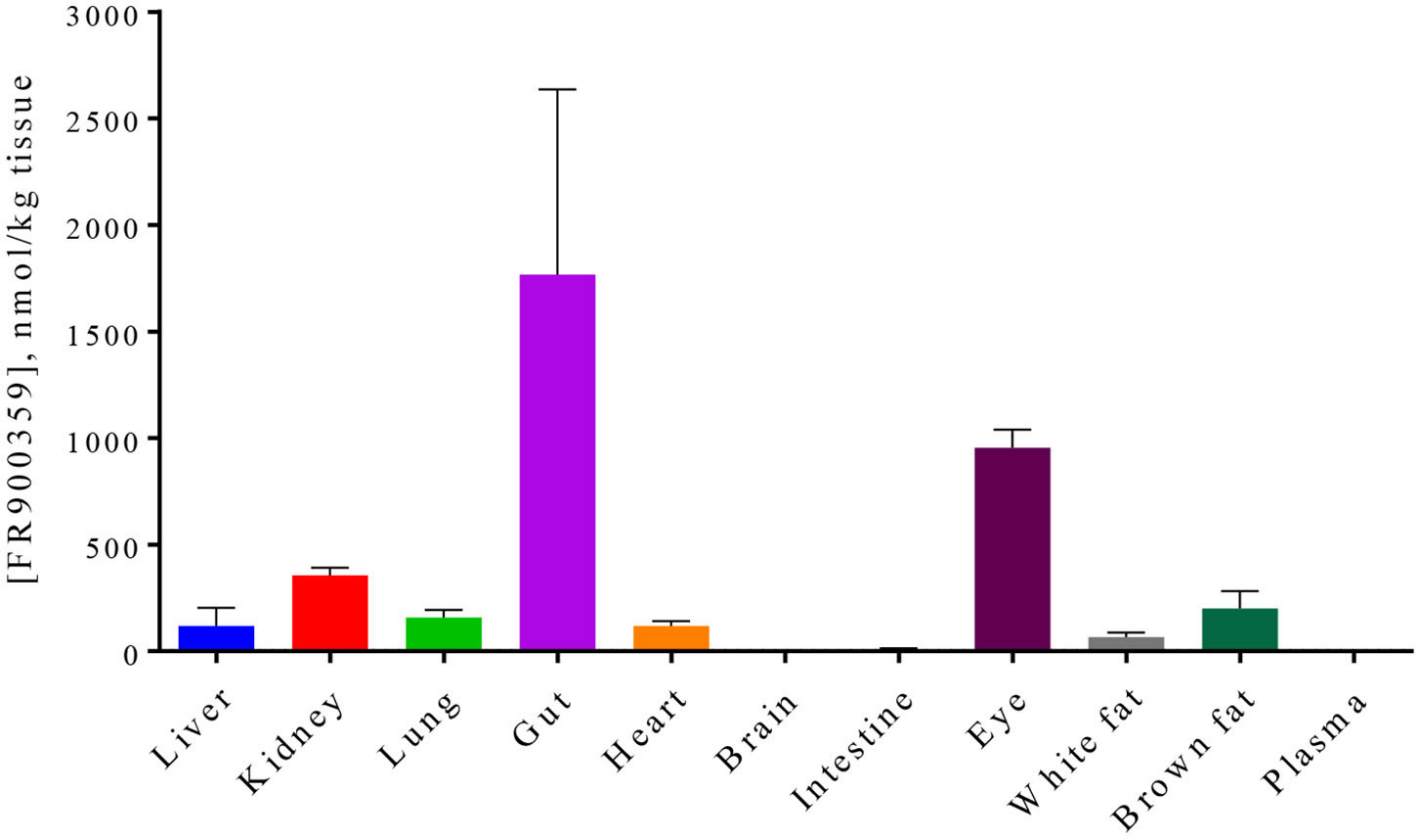
Concentration of FR900359 ± SEM in various tissues after oral application of 0.2 mg FR900359 in mice. Animals were sacrificed after ~1.25 h and organs as well as plasma were harvested and snap-frozen. FR900359 levels in organs from three mice were determined.

The optimized extraction method was subsequently applied to tissue samples from mice treated with a low dose of FR. Approximately 1.25 h after peroral application of 0.2 mg FR, the highest concentrations of FR were detected in the stomach, as expected, and in the eyes. Low levels were found in kidney, lung, heart, liver, and adipose tissue. In intestine, blood plasma, and brain almost no FR was detectable (Figure 4). These results indicate only moderate peroral absorption of FR. Thus, FR might be used for local application which would reduce a high risk of systemic effects.

## Conclusions

The developed quantitative analytical LC-MS/MS method allows sensitive detection of the macrocyclic Gq protein inhibitors FR and YM in (sub)nanomolar concentrations. The established extraction method followed by LC-MS/MS analysis of FR led to highly reproducible results with adequate recovery rates from mouse plasma and tissues, and can likely be applied to other species as well including humans. Our results provide preclinical data for FR as a basis for further research and development of this promising Gq protein inhibitor.

## Data Availability Statement

The raw data supporting the conclusions of this article will be made available by the authors, without undue reservation.

## Ethics Statement

The animal study was reviewed and approved by Landesamt für Natur, Umwelt und Verbraucherschutz, Nordrhein-Westfalen, Germany.

## Author Contributions

MK developed the extraction method and wrote the first draft. JS analyzed the data, wrote the manuscript together with CM, prepared the figures and tables. MS performed most of the LCMS measurements. SK isolated and purified FR. JV contributed to the extraction of the compounds and to the analytical measurements. AS, MM, DW, and BF performed the animal studies and prepared the biological samples. GK supervised the production of FR. CM designed and supervised the study and wrote the final version of the manuscript. All authors contributed to writing of the manuscript.

## Conflict of Interest

The authors declare that the research was conducted in the absence of any commercial or financial relationships that could be construed as a potential conflict of interest.
