# Supplementary material for: Sensitive LC-MS/MS Method for the Quantification of Macrocyclic Gαq Protein Inhibitors in Biological Samples
Source: Front Chem. 2020 Sep 24;8:833. doi: 10.3389/fchem.2020.00833 (PMC7540253; doi:10.3389/fchem.2020.00833)
Supplement: Supplementary file 1 [file Data_Sheet_1.pdf]

Supplementary Information

Sensitive LC-MS/MS method for the quantification of macrocyclic Gα_q protein inhibitors in biological samples

Markus Kuschak[1], Jonathan G. Schlegel[1], Marion Schneider[1], Stefan Kehraus[2], Jan H. Voss[1], Alexander Seidinger[3,4], Michaela Matthey[3,4], Daniela Wenzel[3,4], Bernd K. Fleischmann[3], Gabriele M. König[2], and Christa E. Müller[1]

Affiliations:
[1] PharmaCenter Bonn, Pharmaceutical Institute, Pharmaceutical & Medicinal Chemistry, University of Bonn, Bonn, Germany.
[2] Institute of Pharmaceutical Biology, University of Bonn, Germany.
[3] Institute of Physiology I, Life and Brain Center, Medical Faculty, University of Bonn, Bonn, Germany.
[4] Department of Systems Physiology, Medical Faculty, Ruhr University Bochum, Bochum, Germany.

CORRESPONDENCE:

Prof. Dr. Christa E. Müller
christa.mueller@uni-bonn.de

Table of Contents

Figure S1. Calibration curves p. 2

Table S1. Parameters for LCMS method validation for FR (10-2000 nm) p. 3

Figure S2. Determination of recovery rates p. 4

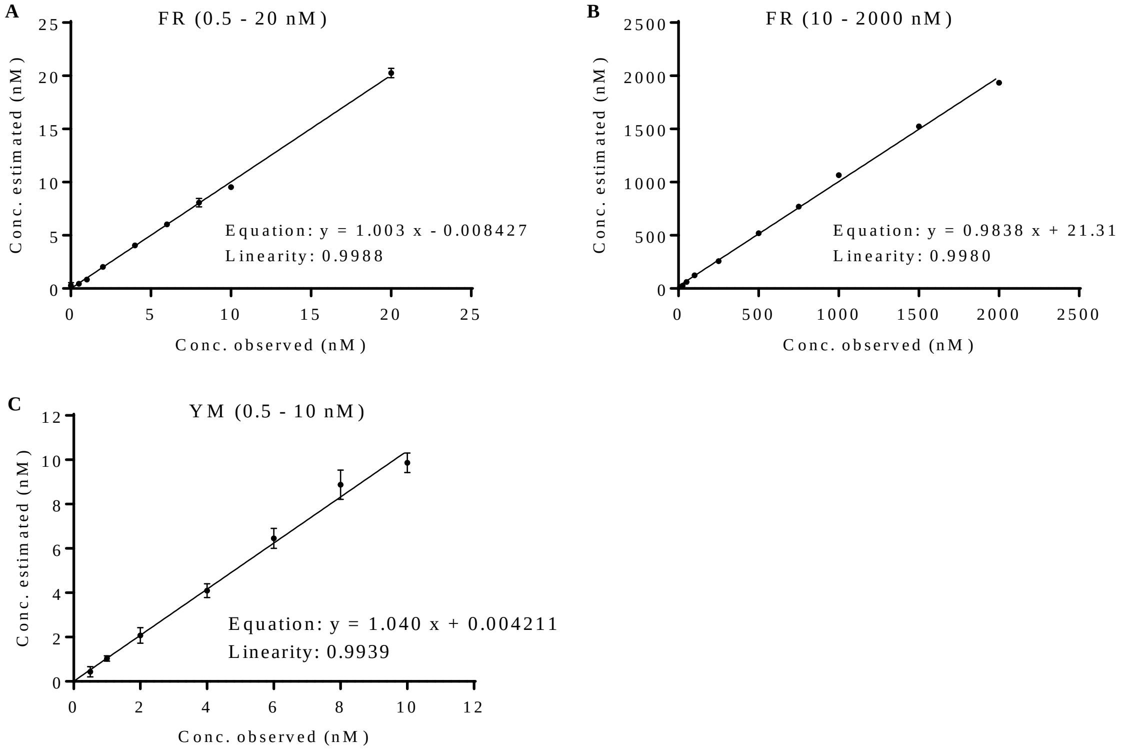

Figure S1. Calibration curves of FR900359 and YM-254890. (**A**) FR900359, 0.5–20 nM, (**B**) FR900359, 10 – 2000 nM, (**C**) YM-254890 0.5 – 10 nM.

Table S1. Results of LC/MS-MS method validation for FR (10-2000 nM)

Parameters	FR900359	Acceptable range[a]
**Regression equation**	y = 0.9838 x + 21.31	
**Linearity** (R^2), n=3	0.9980	
**Accuracy** (recovery; n=3), [%]		
10 nM	108.0	85-115
25 nM	106.7	85-115
50 nM	106.0	85-115
100 nM	106.1	85-115
250 nM	103.2	85-115
500 nM	104.1	85-115
750 nM	102.7	85-115
1000 nM	106.6	85-115
2000 nM	101.6	85-115
**Precision** (RSD; n=3), [%]		
10 nM	2.76	<15
25 nM	6.94	<15
50 nM	2.99	<15
100 nM	6.59	<15
250 nM	3.97	<15
500 nM	2.09	<15
750 nM	0.77	<15
1000 nM	1.50	<15
2000 nM	0.86	<15

[a]According to the FDA guideline “Bioanalytical Method Validation Guidance for Industry”

Determination of recovery rates

Mouse tissues (250 mg each) were mixed with 1 mL of an aqueous solution of 100 nM FR containing 2 mM ammonium acetate and 0.1% formic acid. Then, the exactly same workup steps were performed as described in section 2.2 of the manuscript. For plasma recovery rates, 100 µL of plasma was mixed with 1 mL of an aqueous solution of 100 nM FR containing 2 mM of ammonium acetate and 0.1% formic acid, and the mixture was subsequently centrifuged for 20 min at 15,000 *g*. The obtained supernatant was then treated in the same way as described for tissue samples. A recovery rate of 100% would thus correspond to a measured concentration of 1000 nM FR.

The recovery rates for FR from different organ and tissue samples and from blood plasma are shown in Fig. S2.

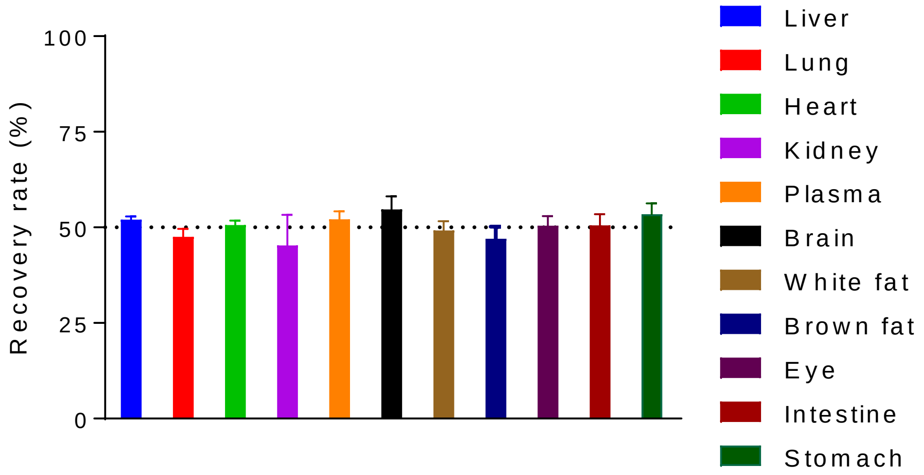

Figure S2. Recovery rate of FR900359 from various mouse tissues and blood plasma. Values represent means ± standard error of the mean (SEM) from three independent experiments.

According to our experience, determination of recovery rates should be repeated for each series of experiments and analytical measurements. To obtain most reliable results, the amount of FR used for spiking should be adjusted with respect to the tissue weight and the FR concentration present in the tissue. Experienced experimentalists can thus get well reproducible recovery rates as shown in Fig. S2.
